# Magnetic resonance imaging characteristics predict pituitary function in non-functional pituitary macro-adenoma undergoing trans-sphenoidal surgery

**DOI:** 10.1186/s12880-022-00787-5

**Published:** 2022-04-01

**Authors:** Behrooz Hassani, Nahid Hashemi-Madani, Manizhe Ataee Kachuee, Mohammad E. Khamseh

**Affiliations:** 1 Department of Radiology, Firouzgar Clinical Research Development Center(FCRDC), Firouzgar General Hospital, Iran University of Medical Sciences(IUMS), Valadi Street, Valiasr Sq., Tehran, Iran; 2 Endocrine Research Center, Institute of Endocrinology and Metabolism, Iran University of Medical Science (IUMS), Tehran, Iran

**Keywords:** Pituitary gland function, Non-functional pituitary macroadenoma, Surgery, Imaging features, Magnetic resonance imaging

## Abstract

**Introduction:**

Maintaining the pituitary function after surgery is highly important. The aim of this study was to investigate the relationship between preoperative magnetic resonance imaging (MRI) characteristics and pituitary function after surgery of non-functional pituitary macroadenoma.

**Methods:**

This retrospective study was performed between 2016 and 2018. Preoperative and postoperative MRI imaging data were retrieved from electronic registration system. The relationship between preoperative MRI characteristics and postoperative pituitary function as well as reconstruction of pituitary gland was investigated using regression models.

**Results:**

Complete data were available for 44 patients. Before surgery, invisible normal tissue was observed in 23 patients (53.5%). Suprasellar extension and cavernous sinus invasion were seen in 36 patients (each one 49.1%). There was a significant reverse relationship between preoperative tumor size and postoperative thyroid stimulating hormone (TSH) (odds ratio (OR): − 0.99 (− 0.18, − 0.003), *p* = 0.04). In addition, we found a significant positive correlation between prolactin level after surgery and tumor size before surgery, (OR: 5.29 (1.65, 8.92), *p* = 0006). Moreover, postoperative panhypopituitarism was observed in 25% of patients with complete morphologic reconstitution of pituitary tissue. While the rate was 50% in patients with no or partial morphologic reconstruction of pituitary tissue.

**Conclusion:**

Preoperative MRI characteristics predict TSH and prolactin level after operation. Furthermore, the adenoma size and volume prior to surgery are the main determinants of normal morphologic reconstruction of pituitary gland.

## Introduction

Non-functional pituitary macroadenomas (more than one centimeter in diameter) comprise about one-third of all pituitary adenomas and are managed with trans sphenoidal surgery [1, 2]. Restoration of normal pituitary function after surgery is important and has been reported in various studies from 20 to 50% [3, 4]. Panhypopituitarism, deficiency in production of at least two pituitary hormones, is a most common complication after pituitary surgery [5]. Inadequate secretion of luteinizing hormone (LH) and follicle stimulating hormone (FSH), growth hormone (GH), thyroid stimulating hormone (TSH) and adrenocorticotropic hormone (ACTH) were observed after pituitary surgery in 99%, 98.6%, 96% and 81.8% of cases after pituitary surgery [6, 7]. Methods for predicting the possible occurrence of these complications have also been studied. Various studies have shown that some findings in magnetic resonance imaging (MRI) are associated with post-operative complications. Therefore, identification of these findings in patients' MRI can be helpful in prediction and, possibly, prevention of the related complications [8, 9]. Researches evaluated the post-operative MRI characteristics over one year after trans sphenoidal surgery of the non-functional pituitary macroadenomas. The results showed the residual of pituitary adenoma in 18% of patients at 3 months after surgery [10, 11]. On the other hand, since the pituitary gland plays an essential role in the control of other endocrine glands, maintaining the function of this gland after surgery is very important and determinating the amount of the normal pituitary tissue after surgery can be very helpful in predicting of the reversibility of pituitary function [12, 13]. Due to the fact that MRI is the method of choice for imaging pituitary tumors and evaluation of changes in the tumor after surgery, MRI before and after surgery is necessary for selection of proper treatment and appropriate postoperative management [12]. Therefore, the aim of this study was to investigate the relationship between pituitary function after trans sphenoidal surgery and MRI characteristics in non-functional pituitary macroadenoma.

## Method

### Study design and data collection

This study was a retrospective cohort study which included patients with non-functional pituitary macroadenoma registered at Iran Pituitary tumor registry (IPTR) between 2016 and 2018. The written informed consent was obtained from the participants and the study was approved by the Ethical Committee at Iran University of medical sciences. (Approval number: IR.IUMS.FMD.REC.1398.154).

### Patients

This study included patients with non-functional pituitary macroadenoma who underwent trans sphenoidal surgery. Inclusion criteria were all patients with biochemically and histologically confirmed non-functional pituitary macroadenoma with available MRI scan who had hormone profile before and after surgery. Patients with a history of radiotherapy as well as those with repeated pituitary surgery were excluded from the study.

### Data collection

All demographic, clinical, and biochemical information were extracted from the electronic record system at the Institute of Endocrinology and Metabolism affiliated to Iran University of Medical Sciences. Hormone profile before surgery and at the last follow-up visit were used in this analysis.

The pre-operative MRI scan as well as the first post-operative MRI scan obtained during 3 months after surgery were also reviewed using the picture archiving and communication system (PACS). The required information about size, volume, consistency and direction of mass displacement were evaluated by two expert radiologists. Size was obtained by measuring the tallest tumor diameter in coronal view. Macroadenoma was defined as greatest tumor diameter greater than 10 mm.

Adenoma volume was calculated using the largest anterior-posterior, external and rostrocaudal radius dimensions. To measure the consistency of macroadenoma, the signal intensity of macroadenoma and that of pons were calculated with quantitative analysis of MRI signal intensity. Then, the consistency was estimated in each case by calculating the ratio of signal intensity of adenoma to pons according to cut point of 1.49 in previous studies; consistency of less than 1.49 was considered as firm and fibrous and more than 1.49 as soft tumors [13]. In this study we used Knosb grading method for assessment of cavernous sinus invasion. Normal gland locations were demonstrated against the pituitary adenoma according to degree of enhancement on preoperative dynamic MR images. After surgery, the normal gland differentiation from the residual enhancing lesion were diagnosed by analyzing the preoperative location and degree of enhancement on dynamic MR imaging and also the position of hypophysial stalk in some cases. Normal hypophysial tissue enhancement is more than adenoma in overall.

### Statistical analysis

The continuous and discrete variables are described using number (percent) and median [interquartile range (IQR)], respectively. To measure the impact of the preoperative MRI findings (tumor size, tumor volume, apparent normal pituitary tissue, and cystic change) on the pituitary function after surgery (TSH, ACTH, LH, GH, and PRL), the univariate regressions models were fitted. Moreover, to assess the MRI characteristics of patients with preoperative pituitary macroadenoma as predictors of normal pituitary gland normal residual pituitary gland (NRPG) after surgery, the univariate logistic regression models were fitted and odds ratios (ORs) were reported. The analyses were performed using the statistical software Stata (ver. 12). The significance level was set to be 0.05.

## Results

Data were available for 44 patients who met the inclusion criteria. Demographic and clinical characteristics of the participants at the time of diagnosis are demonstrated in Table 1.

**Table 1:** Demographic and MRI characteristics prior to surgery

Variables	Prevalence
Age ( yrs)	55 (38–61)
Male (%)	27/44 (61.4%)
Duration of follow-up (mos.)	6.8 (3.6–10.9)
Tumor size (mm)	28 (22–33)
Tumor volume (mm^3^)	6845.5 (4041.2–1034.9)
Giant adenoma (%)	3/44 (6.8%)
*Apparent normal pituitary tissue (%)*	
No visible tissue	23/43 (53.5%)
Normal	7/43(16.3%)
Distorted	13/43 (30.2%)
Cystic change (%)	15/42 (35.7%)
*Tumor extension (%)*	
Suprasellar	18/43(41.9%)
Cavernous sinus	18/43(41.9%)
infrasellar	7/43(16.3%)
*Consistency of tumor (%)*	
Fibrotic	3/43(7%)
Soft	40/43(93%)

Yrs.; years, mos.: months, mm; millimeter, mm^3^; cubic millimeter, data are presented as number (%), median and interquartile range (IQR)

**Fig. 1:**
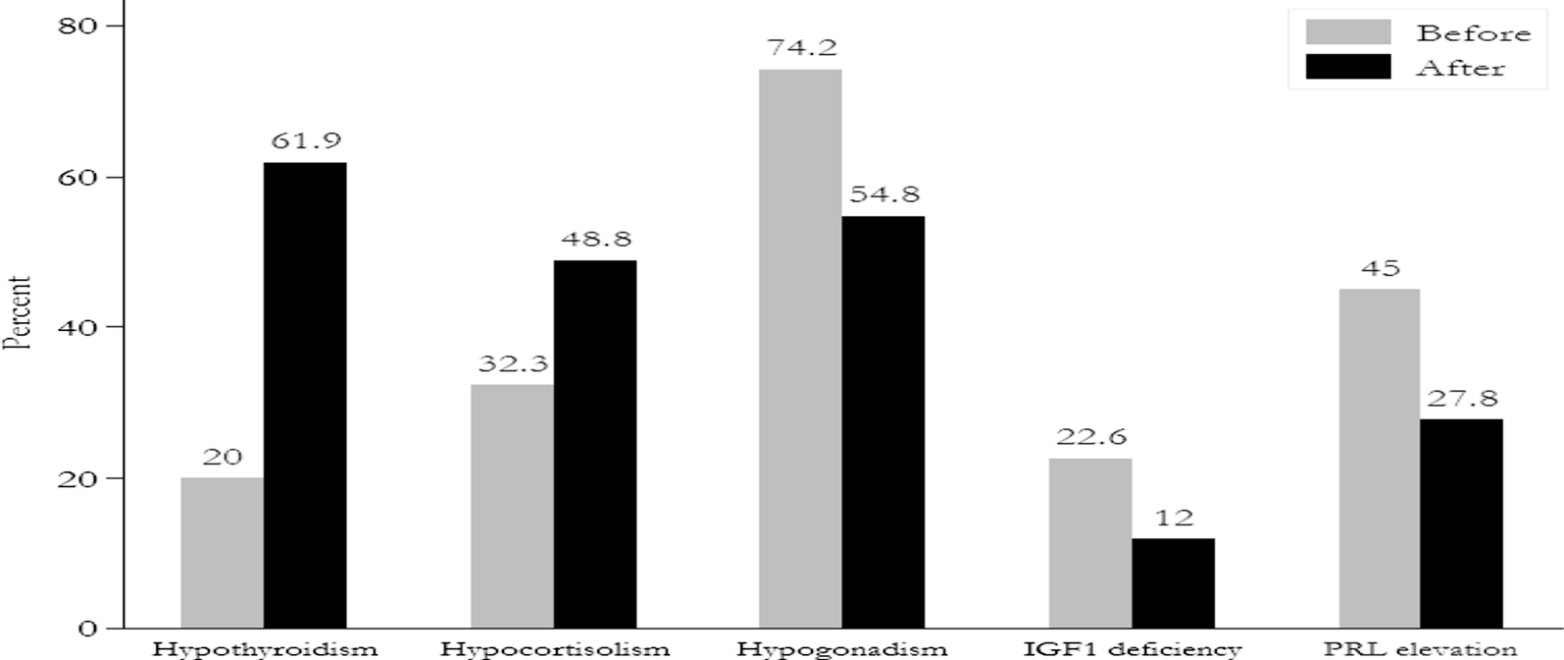
Bar chart of hormonal changes before and after surgery [Insulin-like growth factor-1 (IGF-1), Prolactin (PRL)]

Hypothyroidism was detected before and after surgery in 20% and 61.9% of patients, respectively. Hypercortisolism was diagnosed in 32.3% of patients before and in 48.8% after surgery. Hypogonadism was also observed in 74.2% of patients before and 54.8% of patients after surgery. Low insulin-like Growth Factor-1 (IGF-1) was observed in 22.6% and 12% of patients before and after surgery, respectively. Moreover, hyperprolactinemia was detected in 45% of patients before surgery and in 27.8% of patients after surgery (Fig. 1).

### Preoperative MRI characteristics and postoperative pituitary function

There was a significant, adverse relationship between preoperative tumor size and serum TSH level after surgery (odds ratio (OR): − 0.99 (− 0.18, − 0.003), *p* = 0.04). There was also a significant relationship between preoperative tumor size and postoperative prolactin level (OR: 5.29 (1.65, 8.92), *p* = 0.006). Moreover, a significant relationship was observed between preoperative tumor volume and postoperative prolactin level (OR: 0.006 (0.0008, 0.01), *p* = 0.02) (Table 2). There was no significant relationship between preoperative imaging characteristics and the amount of other pituitary hormones after surgery (*p* ≥ 0.05).

**Table 2:** Relationship between preoperative MRI findings and pituitary function after surgery

Variables	TSH Coefficient (95%CI)	ACTH Coefficient (95%CI)	LH Coefficient (95%CI)	GH Coefficient (95%CI)	PRL Coefficient (95%CI)
Tumor size	− 0.09 (− 0.18, − 0.003) *p* = 0.04	0.36 (− 1.6,2.32) *p* = 0.6	− 0.2 (− 0.42, 0.0091) *p* = 0.06	− 0.005 (− 0.52, 0.4) *p* = 0.8	5.29 (1.65, 8.92) *p* = 0.006
Tumor volume	− 0.0001 (− 0.0002, 0.00001) *p* = 0.08	0.001 (− 0.002, 0.004) *p* = 0.43	− 0.0002 (− 0.0005, 0.00005) *p* = 0.11	− 6.40 (− 0.0008, 0.00007) *p* = 0.8	0.006 (0.0008,0.01) *p* = 0.02
Apparent normal pituitary tissue	− 1.04 (− 2.7, 0.65) *p* = 0.23	11.06 (− 67.74, 13.13) *p* = 0.15	− 1.61 (− 5.64, 2.42) *p* = 0.41	− 0.14 (− 0.95, 0.67) *p* = 0.72	37.44 (− 39.13, 114.01) *p* = 0.3
Cystic change	0.45 (− 1.41, − 2.3) *p* = 0.61	2.67 (− 53.73, 59.06) *p* = 0.91	− 0.55 (− 5.5, 4.4) *p* = 0.82	− 0.32 (− 1.2, 0.58) *p* = 0.5	− 36.4 (− 117.8, 44.88) *p* = 0.37

*CI* confidence interval, *TSH* thyroid-stimulating hormone, *ACTH* adrenocorticotropic hormone, *LH* luteinizing hormone, *GH* growth hormone, *PRL* prolactin

### Preoperative MRI characteristics and postoperative morphologic reconstruction of pituitary gland

**Morphologic** Reconstruction pattern of pituitary gland after surgery was as followed: invisible normal pituitary tissue in 21.4% (9/42) of patients, complete normal pituitary tissue in 45.3% (19/42), and partial remnants of normal pituitary tissue in 33.3% (14/42) patients. There was a significant, adverse relationship between tumor size before surgery and normal morphologic reconstruction of pituitary gland after surgery (OR: 0.80 (0.68, 0.94), *p* = 0.007). There was also a significant, adverse relationship between tumor volume before surgery and NRPG after surgery (OR: 0.99 (0.99, 0.99), *p* = 0.007) (Table 3).

**Table 3:** Preoperative MRI characteristics and odds of the normal residual pituitary gland (NRPG) after surgery

Variables	OR (95% CI)	*p* Value
Tumor size	0.80 (0.68,0.94)	0.007
Tumor volume	0.9998 (0.9996,0.9999)	0.007
Cystic change (No as Ref.)	1.71 (0.30,9.87)	0.546
Tumor extension (Cavernous as Ref.) versus (Supra or Infra)	4 (0.8376,19.1022)	0.082

*NRPG* normal residual pituitary gland

### Pituitary function after surgery and NRPG in MRI scan

**Fig. 2:**
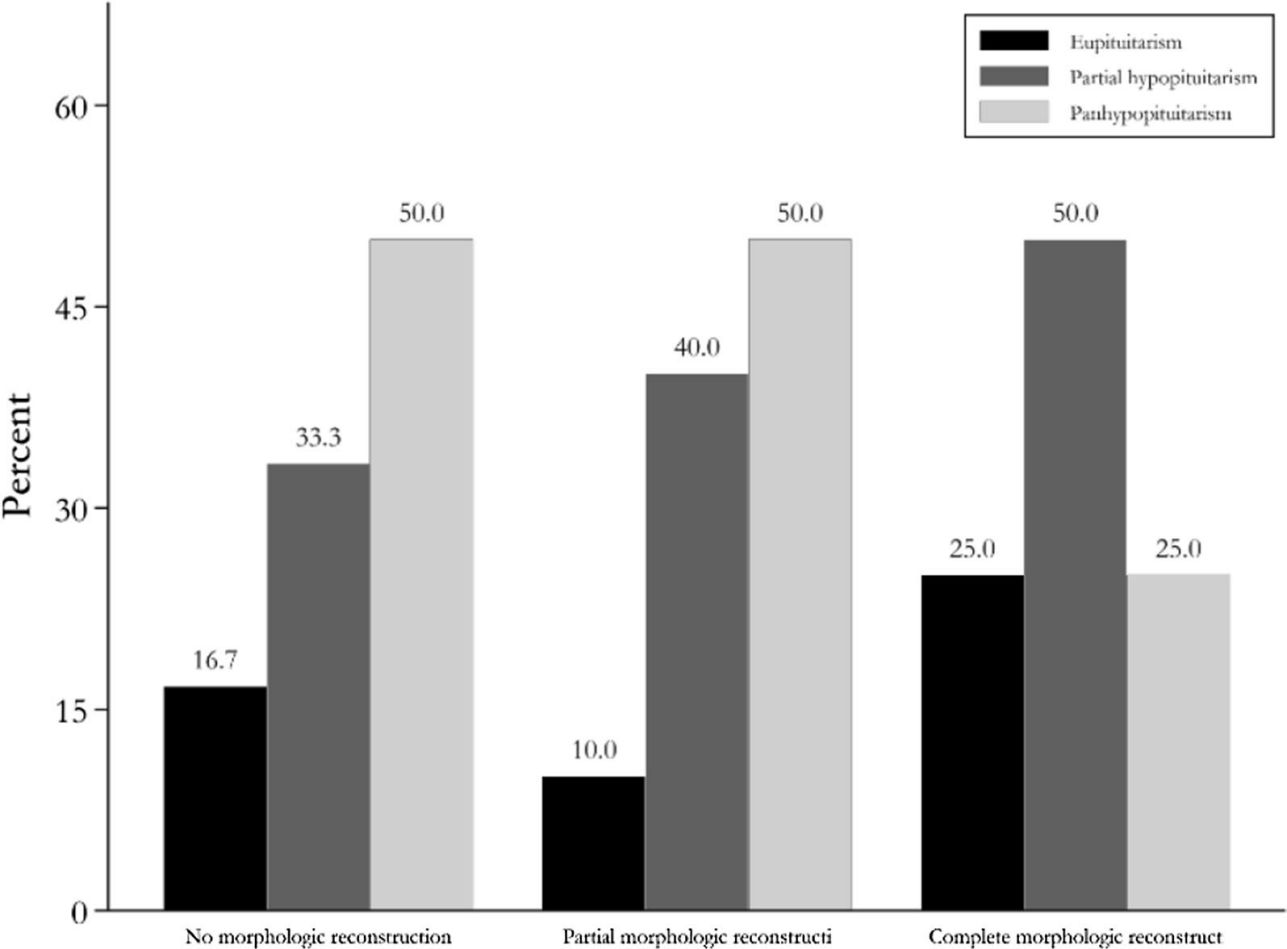
Ratio of patients with different pituitary functions according to the type of morphologic reconstruction of normal pituitary gland on MRI after surgery

Among patients with no visible pituitary tissue (no morphologic reconstruction), normal pituitary function, partial hypopituitarism and pan hypopituitarism were observed in 16.7%, 33.3% and 50% of patients, respectively. In patients with partial morphologic reconstruction of pituitary tissue, these features were observed in 10% (1 patient), 40% (4 patients) and 50% (5 patients), respectively. The patients who had complete morphologic reconstruction of pituitary tissue experienced normal pituitary function (25%), partial hypopituitarism (50%) and panhypopituitarism (25%) (Fig. 2).

## Discussion

MRI is universally used in postoperative care pituitary adenomas, for the diagnosis of residual or recurrent tumors [11, 12]. This retrospective cohort study conducted among 44 patients who underwent TSS due to non-functional pituitary macroadenoma indicated post-operative TSH and prolactin levels are more likely to be associated with some MRI characteristics before surgery namely size and volume of the tumor. Moreover, size and volume of tumor in MRI images before trans sphenoidal surgery (TSS) showed an adverse association with the percentage of normal residual of pituitary gland. In the conducted study by Di Maio et al. in 2012, NRPG was identified in 79% of the patients on preoperative MRI [12]. Our research showed that NRPG was diagnosed in 21.4% of the patients on postoperative MRI, and there was a strong significant relationship between the size and volume of the tumor before surgery and NRPG. MRI with or without administration of gadolinium contrast agent also allows accurate assessment of the position and function of the tumor before and after surgery [12, 13]. But gadolinium uptake significantly improves diagnosis of the pituitary gland, especially in severe deformity cases of the pituitary gland. Improved diagnosis of the pituitary gland, after gadolinium uptake is due to the rapid and pronounced contrast of the pituitary gland, which is more than the adenoma [12].

In the study performed by Nomikos et al. in 2004, 721 patients with non-functional macroadenoma pituitary surgery were evaluated. 24.4% of patients had hypothyroidism after surgery, the prevalence of which was lower than our study (61.9%). Also in the Nomikos study, 1 year after surgery, preoperative prolactin levels were slightly increased in 25.3% (only 5 patients); increasing preoperative and postoperative prolactin was observed in 45% and 27.5% of patients in the present study, respectively; that was more that Nomikos study [14].

In 2015, Lee et al. conducted a study on 45 patients undergoing sphenoid surgery. In this study, hypogonadism was defined as total serum testosterone level < 4.2 ng/ml. Tumor volume was calculated based on MRI images before and after surgery. Examination of the MRI results showed that the need for long-term postoperative testosterone replacement was significantly associated with a larger volume of preoperative tumor and preoperative testosterone levels. Preoperative tumor volume and testosterone levels affect postoperative hypogonadism. By measuring tumor volume and testosterone levels, surgeons will be able to predict postoperative hypogonadism and the need for long-term hormone replacement [15].

In 2021, Ono et al. examined the clinical features and surgical outcomes of 79 patients with dysfunctional pituitary adenoma. Reduction of growth hormone levels was observed in 37.7% of patients. In our study, also a deficiency of postoperative IGF-1 was observed in 12% of patients, which indicated a lower prevalence of this disorder in the present study. Ono et al. also showed that, hypogonadism after surgery was developed in 19% of patients, but in our study, the prevalence of hypogonadism was 54.8% postoperatively, which was higher than the Ono et al. results. A deficiency of TSH was observed in 6.3% of patients in the Ono et al. results, deficiency of TSH was 61.9% in patients studied in our research, and this matter indicates the higher prevalence of this disorder in our study with compared with Ono et al. [16] results.

In other hand, one of the most common symptoms of dysfunctional pituitary macroadenomas is hypogonadism, which may require long-term hormone replacement. In addition, reducing the pressure on the normal pituitary gland may lead to postoperative recovery of the pituitary gland [15, 17].

The rate and type of changes in hormone’s level after surgery can be different because pituitary function and preservation of normal pituitary tissue after surgery depends on several factors such as tumor characteristic, the amount of pressure caused by the tumor on normal pituitary tissue, the type of surgery selected (for example, in patients undergoing cranial surgery, the incidence of this deterioration is significantly higher) and the age of patients [7, 13, 18, 19].

For example in study performed by Jahangiri et al. in 2016, patients with preoperative endocrine deficits (n = 153, 50%) were significantly older (mean age 60) and had larger adenomas. Postoperative endocrine deficits occurred in 42 (13.7%) patients (Thyroid axis 3%, cortisol axis 6%, and GH/IGF-1 axis 4%). In our study, decreased cortisol was observed in 32.3% and 48.8% of patients before and after surgery, respectively and a deficiency of postoperative IGF-1 was observed in 12% of patients. In similar to Jahangiri et al. [20] results, deficits were occurred in hormones production. Disorders of cortisol levels can also vary before and after pituitary surgery. For example, in patients who have had low blood pressure during anesthesia, or who have been taking medications such as phenytoin, ketoconazole, corticosteroid, and narcotics, the disorder is more pronounced [21, 22].

Guinto-Nishimura et al. in 2020 performed preoperative MRI in patients with non-functional pituitary macroadenoma to determine tumor severity alone and in association with the right cerebellar peduncle in 26 patients. Tumor consistency was assessed as soft in 20 (76.92%) patients. In the present study, 93% of the tumors had a soft consistency, which was higher than the Guinto-Nishimura study. Guinto-Nishimura et al. also showed that preoperative tumor volume was 28.7 ± 26.3 cm^3^ and the rate of tumor resection was not significantly associated with tumor consistency [23].

Onofrj et al. in 2018 reported that the mean preoperative tumor volume was 24.66 cm^3^. A progressive tumor volume decrease was noted during follow-up, and symptoms improved in 78% of patients. They conducted the duration of symptoms prior of surgery is a more important factor than tumor resection volume alone when considering the long-term outcome of symptoms. In our study preoperative mean tumor volume was 6845.5 mm^3^ and a significant relationship was observed between preoperative tumor volume and postoperative production of prolactin hormone [24].

Juthani et al. in 2020 performed a study on the 212 patients with pituitary MRI before and after surgery. 62% of patients underwent resection of the tumor based on postoperative MRI findings and comparison with preoperative resection, and as a result, the remnants of the tumor had to be removed. Tumor resection due to postoperative MRI results was significantly associated with increased survival and pituitary function as well as hormone therapy. These results suggest that using preoperative MRI is a safe method leading to the increase in the resection rate of pituitary adenomas. Especially when MRI is combined with endoscopy, it provides the ability to adapt to the removal of tumors while optimizing pituitary function, resulting in a high rate of recovery of secretory hormone [25].

In the results of the present study, normal tissue was not observed in 21.4% of MRI cases after surgery. MRI with or without administration of gadolinium contrast agent also allows accurate assessment of the position of the tumor before surgery and staging of pituitary tumors [13, 23]. Manifestations of normal pituitary gland tissue on MRI images include the size and shape of the gland reflecting pituitary function, which depends on the patient's position, the intensity of the adenohypophysis and neurohypophysis signals [13].

Given the prevalence of pituitary adenomas and especially their non-functional types, often diagnosed at higher stages, pre- and post- operative MRI scan can be used to choose the right treatment approach before.

## Conclusion

There was a significant relationship between preoperative tumor size and volume and postoperative pituitary hormone production. Moreover, preoperative MRI characteristics could predict normal pituitary morphologic reconstruction after pituitary surgery.

## Data Availability

The datasets analyzed during the current study are not publicly available due to limitations of ethical approval involving the patient data and anonymity but are available from the corresponding author on reasonable request.

## Abbreviations

MRI: Magnetic resonance imaging
TSH: Thyroid stimulating hormone
IUMS: Iran University of Medical Science
FCRDC: Firouzgar Clinical Research Development Center
NRPG: Normal reconstruction of pituitary gland
LH: Luteinizing hormone
FSH: Follicle stimulating hormone
GH: Growth hormone
ACTH: Adrenocorticotropic hormone
IPTR: Iran Pituitary tumor registry
PACS: Picture Archiving and Communication System
IQR: Interquartile range
PRL: Prolactin
ORs: Odds ratio
IGF-1: Insulin-like Growth Factor-1
TSS: Trans sphenoidal surgery
